# Oral Adjuvant Curcumin Therapy for Attaining Clinical Remission in Ulcerative Colitis: A Systematic Review and Meta-Analysis of Randomized Controlled Trials

**DOI:** 10.3390/nu10111737

**Published:** 2018-11-12

**Authors:** Maria G. Grammatikopoulou, Konstantinos Gkiouras, Xenophon Theodoridis, Eleni Asteriou, Alastair Forbes, Dimitrios P. Bogdanos

**Affiliations:** 1 Faculty of Medicine, School of Health Sciences, University of Thessaly, Mezourlo, GR41110 Larissa, Greece; xenofontheodoridis@gmail.com; 2 Department of Nutrition & Dietetics, Alexander Technological Educational Institute, Sindos, P.O. Box 141, GR57400 Thessaloniki, Greece; 3 Medical School, Faculty of Health Sciences, Aristotle University of Thessaloniki, University Campus, GR54124 Thessaloniki, Greece; 4 Laboratory of Clinical Pharmacology, Medical School, Faculty of Health Sciences, Aristotle University of Thessaloniki, University Campus, GR54124 Thessaloniki, Greece; kostasgkiouras@hotmail.com; 5 Department of Rheumatology and Clinical Immunology, Faculty of Medicine, School of Health Sciences, University of Thessaly, Biopolis, GR41110 Larissa, Greece; eleniaster91@gmail.com (E.A.); bogdanos@med.uth.gr (D.P.B.); 6 Norwich Medical School, University of East Anglia, Bob Champion Building, James Watson Road, Norwich NR4 7UQ, UK; alastair.forbes@uea.ac.uk; 7 Division of Transplantation Immunology and Mucosal Biology, MRC Centre for Transplantation, King’s College London Medical School, London SE5 9RS, UK

**Keywords:** inflammatory bowel disease, diet, turmeric, proctocolitis, proctitis, ulcerative colitis, diet, curcumin, meta-analysis, RCT, autoimmune disease, gastrointestinal disease, autoimmune diet, rare events, nutraceutical, IBD, beta binomial

## Abstract

Curcumin has demonstrated anti-inflammatory properties and has been investigated as an adjuvant therapy of ulcerative colitis (UC). The scope of this study was to systematically review and meta-analyze the efficacy of oral curcumin administration as an adjuvant therapy of UC. MEDLINE, Cochrane/CENTRAL, ClinicalTrials.gov, WHO-ICT Registry, EMBASE and grey literature were searched for relevant randomized controlled trials (RCTs). The primary outcome was clinical remission (attainment) and the secondary outcome was clinical response (maintenance/failure). Risk of bias was assessed with the Cochrane tool. Odds ratios (OR) were calculated with a Mantel-Haenszel (M-H) random effects model and with a beta-binomial (B-B) random effects model when zero events/cells occurred. Four RCTs met the criteria, but one was removed from the analyses due to inconsistency in protocol details. With the M-H method, treatment with curcumin was significantly superior to placebo in attaining remission in the per-protocol (PP) analysis (OR = 5.83, 95%CI = 1.24–27.43), but not in the intention-to-treat (ITT) analysis (OR = 4.33, 95%CI = 0.78–24.00). However, with the more accurate B-B method, both analyses were insignificant (for PP OR = 4.26, 95%CI = 0.59–31.00, for ITT OR = 3.80, 95%CI = 0.55–26.28). Based on the current available evidence, oral curcumin administration does not seem superior to placebo in attaining remission in patients with UC. Future RCTs should be planned more cautiously with sufficient size and adhere to the ITT analysis in all outcomes.

## 1. Introduction

Inflammatory bowel disease (IBD) is a blanket term used to define chronic inflammatory conditions of the colon and small intestine. IBD’s most typical forms include ulcerative colitis (UC) and Crohn’s disease (CD), both demonstrating an increasing prevalence globally, in particular in Europe and North America [1]. Though the etiology behind IBD development and UC in particular is still uncertain [2], its treatment is based on a comprehensive approach including lifestyle, diet [3,4,5] and prescription of top-down biologic therapy [6]. The main conventional therapeutic targets include immuno-suppression and activation of anti-inflammation pathways [7], with a variety of nutraceuticals having emerged as complimentary pro-active treatment approaches [8].

Among the most promising recent therapeutic advances, curcumin, a polyphenol derived from the turmeric plant (*Curcuma longa*) has been suggested to yield many health-related benefits and pharmacological effects, including its anti-inflammatory [9] and antibacterial actions [10], the ability to improve symptoms of auto-immune diseases [11,12], ameliorate lipidemic profiles [13], and improve vascular endothelial function [14]. Curcumin is accordingly used as a parallel complementary IBD therapy, thought to ameliorate disease related symptoms and maintain remission [15,16]. UC-specific postulated effects of curcumin use include significantly reduced disease activity indices [17,18], lower endoscopic activity scores [15,17], as well as improved erythrocyte sedimentation rate (ESR), a reliable systemic inflammatory marker [17].

Despite the lack of adequate clinical trials, or the publication of relevant systematic reviews [16], adjuvant use of curcumin in UC has become quite common recently [15], being considered by many, as a paradigm shift in complementary therapy of UC. Many narrative reviews and case-control studies speculate on health-related benefits, but the evidence for effectiveness remains vague.

The purpose of this study was to systematically review the literature for randomized control trials (RCTs) evaluating the efficacy of oral curcumin administration, in patients with ulcerative colitis.

## 2. Materials and Methods

### 2.1. Eligibility Criteria and PICO

Eligibility criteria were specified for the search procedure and meta-analysis, using the PICO format (Population (P), Intervention (I), Comparison (C), and Outcomes (O)). The research question was: What is the effect of oral adjuvant curcumin therapy on achieving remission in adult UC patients? The study population was UC (1) adults, (2) with a clinically and endoscopic confirmed diagnosis of UC, (3) having active mild-to-moderate disease activity (defined by a disease-specific activity index) at baseline, while (4) lacking any concomitant diagnosis of an active autoimmune disease, hepatic, renal, or neurological disorder. Intervention involved RCTs comparing oral adjuvant curcumin therapy vs. placebo, or no adjuvant among patients receiving the same pharmacotherapy. The selected primary outcome of the analysis was the proportion of patients attaining remission, as defined by a decreased disease-specific activity score, such as the colitis activity index (CAI) [19], simple clinical colitis activity index (SCCAI) [20], or ulcerative colitis disease activity index (UCDAI) [21]. Secondary outcomes, wherever available for analysis, included (1) clinical response and/or treatment failure, defining remission maintenance (including frequency of adverse effects and changes in disease activity score), (2) mucosal healing (endoscopic remission) as defined by reduction in endoscopic score by either the Baron [22] endoscopic score (BES), or the partial Mayo endoscopic score (MES) [23,24], and (3) time to relapse.

### 2.2. Search Strategy

We performed a comprehensive literature search that included studies from inception until 1 August 2018, using a combination of free-text and MeSH terms. Relevant studies were identified using PubMed/Medline, Web of Science, Cochrane CENTRAL, EMBASE, Clinical Trials, WHO International Clinical Trials Registry, Scopus and Google, for grey literature. Reference lists of included studies were also explored. Only studies published in the English language were selected. The search strategy was based on the Peer Review of Electronic Search Strategies (PRESS) Guidelines [25]. The primary searched terms involved the intervention (curcumin/turmeric use) and the population (ulcerative colitis or IBD patients), in order to identify all types of studies, including reviews which often site additional studies. In more detail, the following keywords: ((inflammatory bowel disease) OR (ulcerative colitis) OR (proctitis) OR (proctocolitis)) AND ((curcumin) OR (turmeric) OR (curcuminoids) OR (*Curcuma longa*)). In a successive search, the use of a sensitivity filter for the identification of RCTs was applied, according to the Cochrane Handbook guidelines [26]. The protocol was registered at PROSPERO (CRD42018098996).

### 2.3. Selection of Studies and Interventions of Interest

Initially, two independent reviewers (M.G.G. and K.G.) identified studies from their titles and abstracts. Full-text articles were retrieved to assist decision-making in cases when deemed necessary. Disagreement between the two reviewers was resolved by a third researcher (D.P.B.). 

RCTs published in the English language were selected when meeting all of the following criteria: (1) having been performed on adult humans, (2) with oral intervention of curcumin vs. either placebo, or no intervention, as an adjuvant therapy. Exclusion criteria involved RCTs performed (1) on animals or (2) children, (3) curcumin intervention delivered in the form of enema instead of *per os*.

### 2.4. Data Extraction

Two reviewers (M.G.G. and K.G.) independently extracted characteristics of the retrieved studies and outcomes of interest. The PRISMA flowchart for systematic reviews was applied to capture the step-by-step exclusion of unrelated/duplicate retrieved records. When disagreement was evident, a third reviewer (D.P.B.) resolved the issue.

Data was extracted from all RCTs in a predefined data extraction form, including study and participant baseline characteristics, intervention details (dose, times per day, duration, form, etc.), comparators, and clinical outcomes to produce summary tables of included studies (adverse events, funding information and discontinued/drop out).

### 2.5. Risk of Bias and Quality Assessment

The Grading of Recommendations Assessment, Development and Evaluation (GRADE) [27] was used by three scientists for appraisal of the retrieved studies. Results were entered in the Cochrane Risk of Bias (RoB) 2.0 tool [26] for presentation of bias in a comprehensive manner. The quality of studies was classified as being at of high, unclear or low risk of bias. Additionally, the Oxford quality scoring system (Jadad score) [28] was used to assess the quality of retrieved RCTs.

### 2.6. Statistical Analyses

Meta-analysis was considered when available information was found from at least three studies, thus it was run only for the primary outcome (remission). Remission was treated as dichotomous variable in the analyses and its available data from the included RCTs were pooled by calculating odds ratios (OR) along with their respective 95% confidence intervals (CI) with the Mantel–Haenszel (M-H) method [29], in a random effects model. Different analyses were performed for intention-to-treat (ITT) and per protocol (PP) data.

Heterogeneity of included data was assessed with the use of the chi-square test and the I^2^ statistic. The estimation of OR with their confidence intervals (CI) and the presence of heterogeneity were analyzed with RevMan as specified in the protocol (Review Manager [Computer program]. Version 5.3. Copenhagen: The Nordic Cochrane Centre, The Cochrane Collaboration, 2014) [30]. However, RevMan applies a continuity correction, by adding a 0.5 value in trials with zero events in a cell, and this analysis has been showed to be biased [26,31]. The analysis of the primary outcome where two studies had zero events in the placebo group was supplemented with the calculation of ORs via a beta-binomial (B-B) random effects model. This analysis was carried out on SAS^®^ University Edition (SAS Institute Inc., Cary, NC, USA) with the macro suggested by Sharma et al. [31]. For inferential reasoning on B-B method of calculation of ORs and CI, non-significant results have the value of 1.0 within their CI range.

## 3. Results

**Figure 1:**
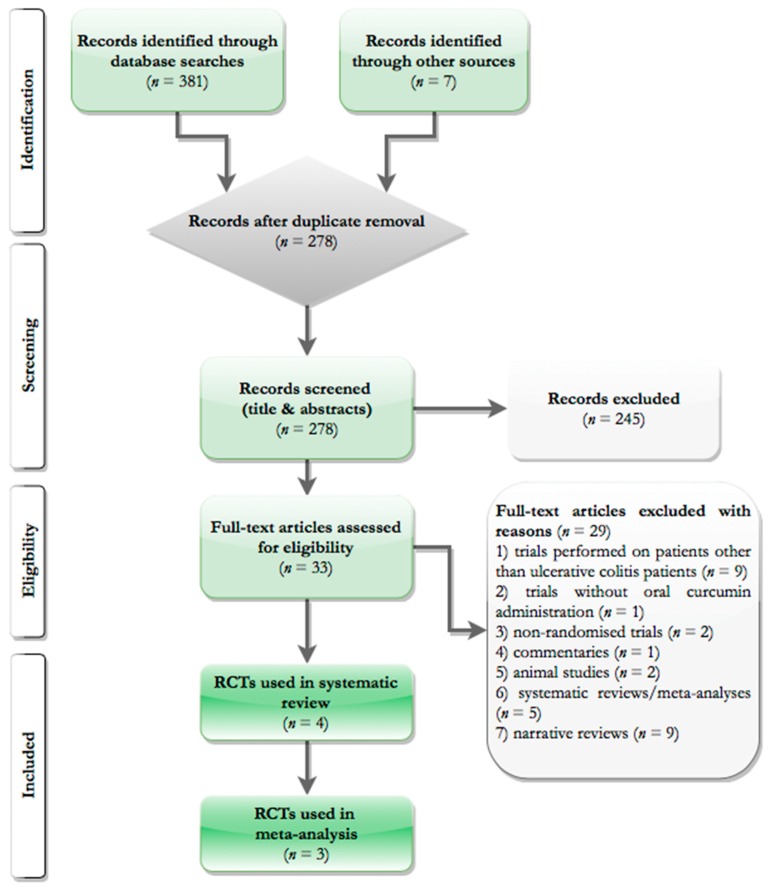
PRISMA flowchart of the selection process.

Figure 1 describes the flow chart of the studies selection process. A total of four RCTs were identified and three were included in the final analyses.

### 3.1. Study Characteristics and Risk of Bias

**Figure 2:**
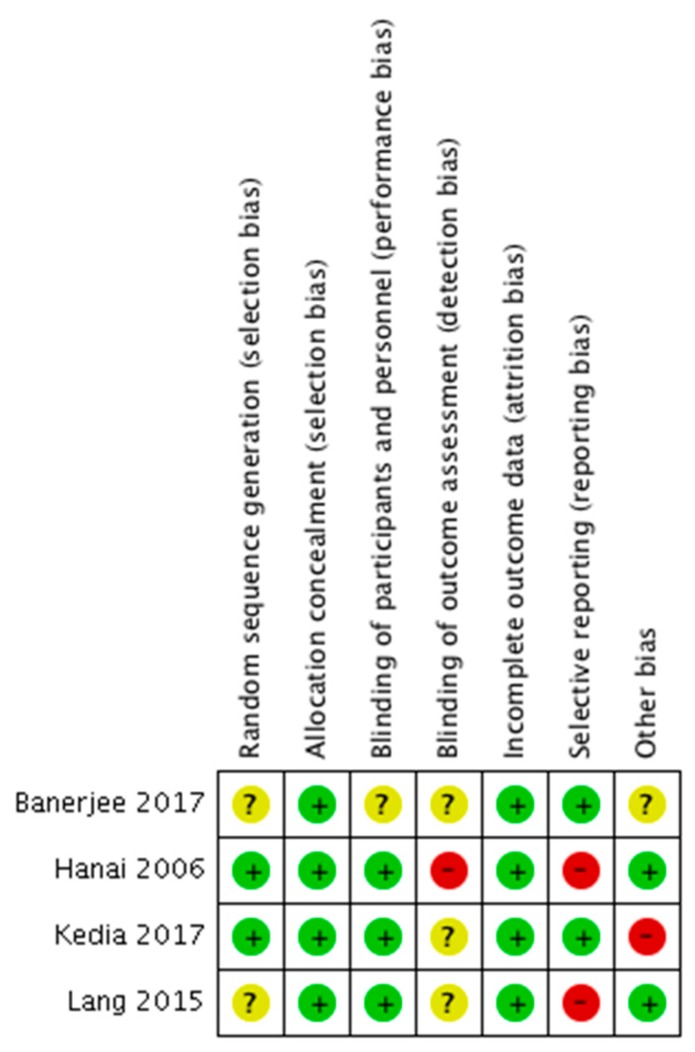
Risk of bias summary, stressing authors’ judgments about each risk of bias item for the included RCTs.

Table 1 details the characteristics and outcomes of each included study. All studies were conducted in outpatient settings using parallel RCT designs. The summary of the Risk of Bias, stressing authors’ judgments about each risk of bias item for the included RCTs, is presented in Figure 2.

### 3.2. Exclusion of RCTs 

The Banerjee trial [34] demonstrated the highest risk of bias and the lowest Jadad quality score. Additionally, several inconsistencies were observed with regards to the trial, between the registered protocol and the results presented at the American Gastroenterology Association Digestive Disease Week. In further detail, discrepancies concerning the duration of the intervention were noted (1 year vs. 3 months), the outcomes and their measures, as well as in the dose of administered curcumin. According to the registry, administered curcumin dose started at 50 mg/day of Self-Micro Emulsifying Drug Delivery System (SMEDDS), increased at 100 mg/day, if lack of response was noted at two weeks’ time. However, in the results, reported administered dose was exactly 50 mg/day, while in parallel, the time intervals for evaluating clinical response did not include the two weeks from trial initiation. Additionally, clinical response was evaluated endoscopically with the MES, and thus referred to endoscopic remission, rather than clinical. In parallel, according to the registry, evaluation of endoscopic remission was reported to be performed using the Ulcerative Colitis Endoscopic Index of Severity (UCEIS), whereas in the published results, the MES was mentioned instead. Due to these observations, we decided to exclude the Banerjee trial [34] from all performed analyses.

### 3.3. Effects of the Intervention on Remission Attainment

**Figure 3:**
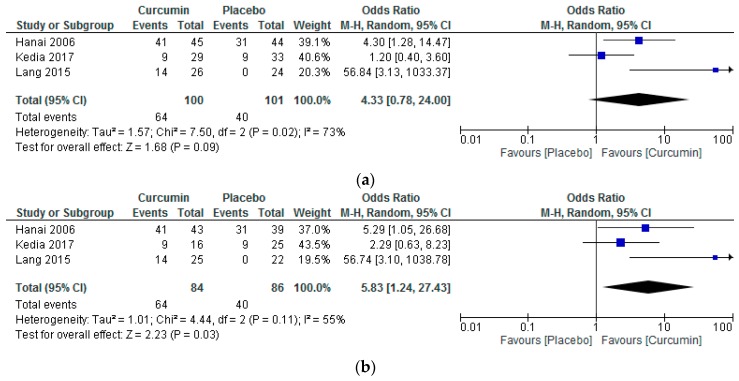
Forest plots for the attainment of remission among ulcerative colitis patients receiving curcumin vs. placebo as an adjuvant according to the intention-to-treat (**a**), and per protocol analyses (**b**). CI: confidence intervals; M-H: Mantel–Haenszel; Random: Random effects model.

According to the M-H method of calculating the OR in RevMan, treatment with curcumin was superior to placebo for achieving remission in the PP analysis of the included studies (Figure 3b, M-H OR = 5.83, 95% CI = 1.24 to 27.43) but not in the ITT analysis (Figure 3a, M-H OR = 4.33, 95% CI = 0.78 to 24.00). However, with the B-B method of calculating the OR, none of the above-mentioned analyses were significant (PP analysis B-B OR = 4.26, 95% CI = 0.59 to 31.00, and ITT analysis: B-B OR = 3.80, 95% CI = 0.55 to 26.28). Furthermore, notable heterogeneity was observed in the ITT analysis (chi-square = 7.50, df = 2, *p* = 0.02; I^2^ = 73%) and a moderate level of heterogeneity in the respective PP analysis (chi-square = 4.44, df = 2, *p* = 0.11, I^2^ = 55%).

### 3.4. Effects of the Intervention on Other Outcomes

Other outcomes, including differences in endoscopic scores and time to relapse, were not meta-analyzed because these were not reported by a minimum of three RCTs, after exclusion of the Banerjee [34] trial.

## 4. Discussion

Our results indicate that based on the synthesis of the currently available evidence, oral adjuvant curcumin therapy does not appear to contribute to attaining remission among adult patients with mild-to-moderate UC. Despite evidence from individual RCTs indicating that curcumin might be beneficial for UC patients, pooled data did not reveal a significant effect.

However, it should be noted that our study is based on three RCTs, and thus the total sample of participants in the analyses was limited, not allowing for subgroup analyses and definite conclusions. Considering that a minimum of two RCTs is considered adequate for performing meta-analyses [37,38], the three trials included in our analysis was considered an adequate sample number for further analysis, nevertheless, results have to be treated with caution. In an effort to increase the number of retrieved studies, we decided to expand our search beyond the protocol registered in PROSPERO, by including searches in more languages, other than English. These additional searches focused mainly on Asian languages (Chinese, Indian, etc.), where the medicinal use of turmeric is traditional, spanning for thousands of years [39]. Unfortunately, this post-protocol search failed to retrieve more RCTs, despite the multiple databases searches (Chinese Clinical Trial Register, Clinical Trials Registry India, Sri Lanka Clinical Trials Registry, IndMED, PakMediNet, IBECS, etc.). An additional factor possibly explaining the lack of a positive effect might stem from the highly heterogenous curcumin doses *per os* observed in the retrieved RCTs, spanning from 450 mg to 3 g daily, while further discrepancies were noted in the definition of the outcomes and the tools used to define them, both reflected in the increased heterogeneity of the pooled analyses. In parallel, we are unaware of the degree of curcumin purity in each of the included RTCs [40], and this might have induced bias in the observed effect of the pooled analyses. Nevertheless, we are waiting for the completion of two ongoing RCTs (NCT02277223 and NCT03122613), which are due by the year 2021.

Additionally, our analysis has inherited all limitations of the included trials; thus, disease extend was not mentioned in either RCTs and it is highly possible that patients have responded differently in curcumin administration in distinct Montreal classifications [35]. An additional limitation for consideration is the timing between completion of the trials and publication, with the Kedia trial [33] being published 12 year post-completion, lacking trial registration and funding reporting for the curcumin and placebo tabs. However, we considered this RCT eligible for the analyses taking into account that (1) it received the highest Jadad score, (2) the publication delay might well have been the result of the reported lag-time observed in the dissemination and publication of research findings [41], and, finally, (3) the RCT was conducted during a time when trial registration was not deemed necessary (circa 2003). As for the inclusion of three RCTs in the present pooled analysis, it is considered adequate in terms of methodology [37,38], with many recent meta-analyses published in high-impact journals synthetizing data from three RCTs [42,43]. While some might argue that a systematic review alone might be more appropriate, it is characteristic, that the median number of studies included in meta-analyses of cardiovascular events published in the Cochrane Reviews is reported to be three [44], whereas even in UC research, recent meta-analyses have pooled three to four RCTs [45]. Although undoubtedly more is better meta-analysis-wise, with three pooled RCTs being considered adequate, researchers should focus more on the methodological quality [46], rather than what is individually perceived as an adequate sample size.

In addition, it should be noted that oral curcumin administration might be inferior compared to the enema administration, as far as UC is concerned, due to the more direct and proximal delivery in the second. As such, RCTs should also explore the administration of curcumin via enema, in order to define the most effective delivery route, since only one RCT [47] has assessed this route to date. Furthermore, taking into consideration that this review has retrieved RCTs with relatively low sample sizes, this could be considered as a primary limitation as for other meta-analyses of the specific research field. This highlights the need for future RCTs to be meticulously planned in advance with sufficient sample sizes, aiming to provide more confident conclusions regarding the effectiveness of curcumin treatment (either via oral, or enema administration).

Our study also verified errors in the significance of pooled analyses with the use of RevMan, in trials with rare events. Verification of the significance via B-B test eliminated the significant outcomes, indicating that RevMan, although popular and user-friendly, has inherited limitations that can easily lead to wrong estimations and subsequently, wrong conclusions regarding a therapy. Overall, software and methods using the continuity correction (addition of 0.5 in rare events) should be avoided [48].

Finally, at the time this manuscript was drafted, a meta-analysis on the same topic has appeared in press [49], publishing pooled results from three RCTs (142 UC patients in total), while reporting that the use of curcumin along with mesalamine was associated with increased odds of clinical remission. Overall, a tendency towards clinical improvement, endoscopic remission and improvement rate was also noted in the curcumin group compared to placebo. The fact that this study did not include all RCTs that we retrieved, while the authors did not control for the oral administration of curcumin only (one of the three studies included involved enema administration), pooling heterogeneous trials together, while unveiling methodological pitfalls that we avoided. Notably, those authors [49] did not control for the false-significant results appearing when rare events exist in the included RCTs, which further underlines the lack of proper assessment.

## 5. Conclusions

In conclusion, the present analyses showed that based on the current available evidence, oral adjuvant curcumin therapy does not appear to contribute to either attaining remission, or ameliorating clinical response among patients with UC. However, absence of evidence is not evidence of absence and, given the heterogeneity demonstrated herein, and the fact that curcumin research is still in the pipeline, caution in needed in data interpretation. Further, well-designed, standardized, long-term RCTs are required, with adequate sample size, rigorous intention-to-treat analyses, sufficient doses and less bias, in order to add weight to the current analyses, produce more robust results and guide clinicians towards/against curcumin supplementation in patients with UC.

## Figures and Tables

**Table 1:** Characteristics and outcomes of the included randomized controlled trials (RCTs).

First Author:	Hanai [17]	Lang [32]	Kedia [33]	Banerjee [34]
Duration:	2004–2005	2011–2014	2003–2005	2016–2017
Countries:	Japan	Israel, Cyprus, Hong Kong	India	India
Registry:	-	NCT01320436	-	NCT02683733
Ethical Approval:	√	√	√	NR
Multicentre:	√	√	-	-
Masking:	Double-blind	Double-blind	Double-blind	Triple-blind
Results publication:	*Clin. Gastroenterol. Hepatol.* (Journal)	*Clin. Gastroenterol. Hepatol.* (Journal)	*World J. Gastrointest. Pharmacol. Ther.* (Journal)	AGA 2017 DDW (abstract published in *Gastroenterol.* Journal)
Timing of publication:	2006	2015	2017	2017
Publication form:	Full-text	Full-text	Full-text	Abstract only
Patients:	CAI ≤ 4 (stable previous 4 weeks) *	Mild–moderate UC (5 ≤ SCCAI < 12)	Mild–moderate UC (3 ≤ UCDAI < 9)	Mild–moderate UC (2 ≤ MES < 6)
Age (years):	13–65 as inclusion, 18–75 as PP	18–70	≥18	18–70
Disease extent (Montreal Classification) [35]:	NR	NR	NR	NR
Treatment:	SZ (1–3 g/day) or Mesalamine (1.5–3 g/day)	Mesalamine oral 4 g/day + topical enema	Mesalamine 2.4 g/day	Mesalamine (oral + enema) with physician defined dose
Inclusion criteria:	√	√	√	√
Exclusion criteria:	√	√	√	√
Treatment arm:	*n* = 43 on curcumin	*n* = 26 on curcumin	*n* = 29 on curcumin	*n* = 22 on curcumin
Control arm:	*n* = 39 on placebo	*n* = 24 on placebo	*n* = 33 on placebo	*n* = 25 on placebo
Intervention:	2 g curcumin/day	3 g curcumin/day	150 mg purified curcumin × 3/day	Registry: 50 mg SMEDDS caps increased to 100 mg after 2 weeks, if no response was noted Results: 50 mg SMEDDS caps
Comparator:	Placebo	Placebo	Placebo	Placebo
Duration:	6 months	1 month	8 weeks	Registry: 1 year Results: 3 months
Measures:	CAI, EI	SCCAI, MES, Hb, CRP,	UCDAI, BES	MES, UCEIS
Timing of clinical remission evaluation:	At month 0, 2, 4, 6, 12	At week 0 and 4	At week 0, 4, and 8, or as required	At week 0 and 6, and 3 months
Timing of relapse evaluation:	At month 2, 4, 6, 12	At week 4	At week 4 and 8, or as required	At week 6, and 3 months
Timing of endoscopic remission:	At month 0 and 6	At week 0 and 4	At week 0, 4, and 8, or as required	At week 6, and 3 months
Primary outcome:	Clinical remission (CAI ≤ 4) at 6 months	Clinical remission (SCCAI ≤ 2) at 4 weeks	Clinical remission (UCDAI ≤ 2) at 8 weeks	Clinical remission (MES ≤ 1)
Secondary outcomes:	CAI reduction EI reduction	Clinical response (SCCAI ↓ ≥ 3) Endoscopic remission (MES ≤ 1) Hb < 12 g/dL at 4 weeks) elevated CRP (4 weeks)	Clinical response (UCDAI ↓ ≥ 3) Sigmoidoscopic remission (BES = 0/1) Treatment failure (UCDAI ↑ ≥ +3 points, or treatment intolerance)	Clinical response (MES ↓ ≥ 3) Endoscopic remission (MES ≤ 1 per Results, UCEIS < 3 per Registry)
Relapse definition:	CAI ≥ 5	SCCAI ≥ 5	UCDAI ≥ +3 points from baseline	NR
Mucosal Healing ^‡^ definition:	NR	any drop of ≥ 1 in MES	BES of 0/1	any drop of ≥ 1 in MES
Non-compliance definition:	-	-	failure to take ≥ 80% of medication	-
Adverse effects (severe) (*n*):	-	*n* = 3, indifferent between arms (On 1 patient with peptic ulcer before initiation and 2 with worsening UC symptoms)	-	NR
Adverse effects (mild) (*n*):	*n* = 7 Abdominal bulging, (transient hypertension, transient increase in the number of stools, nausea, and elevated γ–guanosine triphosphate levels in a regular alcohol drinker)	*n* = 4, indifferent between arms (mild nausea, transient increase in stool frequency, and abdominal bloating)	-	NR
Discontinuation (*n*):	Treatment arm: 2/45 (1 with hypertension withdrew and 1 received prednisone) Control arm: 5/44 (2 withdrew and 3 received either prednisone, or immunosuppressants)	Treatment arm: 1/26 (with pre-existing peptic ulcer) Control arm: 2/24 (1 lost to follow-up, 1 withdrew consent)	Treatment arm: 13/29 (8 with worsening UC symptoms) Control arm: 8/33 (2 with worsening UC symptoms)	Treatment arm: 3/22 Control arm: 2/25 (No further data were reported)
Clinical remission PP (*n*):	Treatment arm: 41/43 (6 months), 33/43 (12 months) Control arm: 31/39 (6 months), 25/39 (12 months)	Treatment arm: 14/25 Control arm: 0/22	Treatment arm: 9/16 Control arm: 9/25	^†^
Clinical remission ITT (*n*):	Treatment arm: 41/45 (6 months), 33/45 (12 months) Control arm: 31/44 (6 months), 25/44 (12 months)	Treatment arm: 14/26 Control arm: 0/24	Treatment arm: 9/29 Control arm: 9/33	^†^
Improved DAI ^#^ PP (*n*):	NR	Treatment arm: 16/25 Control arm: 3/22	Treatment arm: 6/16 Control arm: 12/25	Treatment arm: 12/19 Control arm: 5/23
Improved DAI ^#^ ITT (*n*):	NR	Treatment arm: 16/26 Control arm: 3/24	Treatment arm: 6/29 Control arm: 12/33	Treatment arm: 12/22 Control arm: 5/25
Mucosal Healing ^‡^ PP (*n*):	NR	Treatment arm: 8/22 Control arm: 0/16	Treatment arm: 10/16 Control arm: 10/25	Treatment arm: 5/19 ^†^ Control arm: 0/23 ^†^
Mucosal Healing ^‡^ ITT (*n*):	NR	4 patients in the intervention and 8 controls did not provide permission for endoscopy	Treatment arm: 10/29 Control arm: 10/33	Treatment arm: 5/22 ^†^ Control arm: 0/25^†^
Baseline DAI (mean ± SD):	Treatment arm: CAI = 1.3 ± 1.1 Control arm: CAI = 1.0 ± 1.1	Treatment arm: SCCAI = 6.5 ± 1.5 Control arm: SCCAI = 7.0 ± 1.8	Treatment arm: UCDAI = 5.2 ± 2.0 Control arm: UCDAI = 5.5 ± 1.9	NR
Post-treatment DAI (mean ± SD):	Treatment arm: CAI = 1.0 ± 2.0 (6 months) Control arm: CAI = 2.2 ± 2.3 (6 months)	NR	Treatment arm: UCDAI = 3.4 ± 3.1 Control arm: UCDAI = 3.8 ± 2.8	NR
Baseline EI (mean ± SD):	Treatment arm: EI = 1.3 ± 0.8 Control arm: EI = 1.3 ± 1.0	Treatment arm: EI = 1.9 ± 0.4 Control arm: EI = 2.1 ± 0.39	NR	NR
Post-treatment EI (mean ± SD):	Treatment arm: EI = 0.8 ± 0.6 (6 months) Control arm: EI = 1.6 ± 1.6 (6 months)	Treatment arm: EI = 1.35 ± 1.19 Control arm: EI = 2.25 ± 0.88	NR	NR
Jadad [28] score	4	4	5	2
Funding:	Eli and Edythe L. Broad Foundation, but placebo and curcumin tabs supplied by API Co, Ltd. (Japan)	Partly by the Talpiot Medical Leadership grant (Sheba Medical Center, Leona M. and Harry B. Helmsley Charitable Trust)	NR, but curcumin and placebo were supplied by Himalaya Drug Company (India)	Asian Institute of Gastroenterology

AGA: American Gastroenterology Association; ASA: Aminosalicylic acid; BES: Baron Endoscopic Score [22]; CAI: Colitis Activity Index [19]; CRP: C-reactive protein; DAI: Disease Activity Index (CAI/SCCAI/UCDAI); DDW: Digestive Disease Week; EI: Endoscopic Index [19]; ITT: Intention-to-treat; MES: partial Mayo Endoscopic Score [23,24]; NR: Not Reported; PP: Per Protocol; SCCAI: Simple Clinical Colitis Activity Index [20]; SD: Standard Deviation; SMEDDS: Self-Micro Emulsifying Drug Delivery System; SZ: sulfasalazine; UC: Ulcerative Colitis; UCDAI: Ulcerative colitis Disease Activity Index [21]; UCEIS: Ulcerative Colitis Endoscopic Index of Severity [36]. * Groups matched for gender, age, UC duration, recurrences during the past 2 years, clinical course, CAI, and EI. ^#^ Improved DAI refers to clinical response. ^‡^ Mucosal healing refers to endoscopic remission, or sigmoidoscopic remission. ^†^ In this trial, remission was only evaluated endoscopically, and thus refers to endoscopic remission (mucosal healing) instead of clinical, which was suggested by the authors, since endoscopic remission endpoints were applied (MES and UCEIS). √ RCTs fulfilling this criterion. - RCTs not fulfilling this criterion. ↑ increment. ↓ reduction.
